# Periappendiceal fat-stranding models for discriminating between complicated and uncomplicated acute appendicitis: a diagnostic and validation study

**DOI:** 10.1186/s13017-021-00398-5

**Published:** 2021-10-13

**Authors:** Hui-An Lin, Hung-Wei Tsai, Chun-Chieh Chao, Sheng-Feng Lin

**Affiliations:** 1 Department of Emergency Medicine, Taipei Medical University Hospital, Taipei, Taiwan; 2 Graduate Institute of Injury Prevention and Control, College of Public Health, Taipei Medical University, Taipei, Taiwan; 3 Department of Emergency Medicine, School of Medicine, Taipei Medical University, Taipei, Taiwan; 4 School of Public Health, College of Public Health, Taipei Medical University, 250 Wu-Hsing Street, Taipei City, 110 Taiwan; 5 Department of Public Health, School of Medicine, College of Medicine, Taipei Medical University, Taipei, Taiwan; 6 Department of Critical Care Medicine, Taipei Medical University Hospital, Taipei, Taiwan; 7 Department of Clinical Pathology, Taipei Medical University Hospital, Taipei, Taiwan

**Keywords:** Acute appendicitis, Complicated appendicitis, Fat stranding, Perforation, Scoring system

## Abstract

**Background:**

Recent studies have reported promising outcomes of non-operative treatment for uncomplicated appendicitis; however, the preoperative prediction of complicated appendicitis is challenging. We developed models by incorporating fat stranding (FS), which is commonly observed in perforated appendicitis.

**Material and methods:**

We reviewed the data of 402 consecutive patients with confirmed acute appendicitis from our prospective registry. Multivariate logistic regression was performed to select clinical and radiographic factors predicting complicated acute appendicitis in our model 1 (involving backward elimination) and model 2 (involving stepwise selection). We compared *c* statistics among scoring systems developed by Bröker et al. (in J Surg Res 176(1):79–83. https://doi.org/10.1016/j.jss.2011.09.049, 2012), Imaoka et al. (in World J Emerg Surg 11(1):1–5, 2016), Khan et al. (in Cureus. https://doi.org/1010.7759/cureus.4765, 2019), Kim et al. (in Ann Coloproctol 31(5):192, 2015), Kang et al. (in Medicine 98(23): e15768, 2019), Atema et al. (in Br J Surg 102(8):979–990. https://doi.org/10.1002/bjs.9835, 2015), Avanesov et al. (in Eur Radiol 28(9):3601–3610, 2018), and Kim et al. (in Abdom Radiol 46:1–12, 2020). Finally, we examined our models by performing the integrated discrimination improvement (IDI) test.

**Results:**

Among enrolled patients, 64 (15.9%) had complicated acute appendicitis. We developed new 10-point scoring models by including the following variables: C-reactive protein, neutrophil to lymphocyte ratio, and computed tomography features of FS, ascites, and appendicolith. A cutoff score of ≥ 6 exhibited a high sensitivity of 82.8% and a specificity of 82.8% for model 1 and 81.3% and 82.3% for model 2, respectively, with *c* statistics of 0.878 (model 1) and 0.879 (model 2). Compared with the model developed by Bröker et al. which included C-reactive protein and the abdominal pain duration (*c* statistic: 0.778), the models developed by Atema et al. (*c* statistic: 0.826, IDI: 5.92%, *P* = 0.0248), H.Y Kim et al. (*c* statistics: 0.838, IDI: 13.82%, *P* = 0.0248), and our two models (IDI: 18.29%, *P* < 0.0001) demonstrated a significantly higher diagnostic accuracy.

**Conclusion:**

Our models and the scoring systems developed by Atema et al. and Kim et al. were validated to have a high diagnostic accuracy; moreover, our models included the lowest number of variables.

**Supplementary Information:**

The online version contains supplementary material available at 10.1186/s13017-021-00398-5.

## Introduction

Differentiating between complicated and uncomplicated acute appendicitis preoperatively is challenging [1–3] and crucial. Early appendectomy for uncomplicated appendicitis has long been recommended to prevent its progression toward rupture [4, 5]. Recent randomized controlled trials [6–9] and meta-analyses [10–12] have reported that the non-operative management of uncomplicated acute appendicitis with antibiotic treatment resulted in satisfactory outcomes. In the recently published 2020 update of the World Society of Emergency Surgery (WSES) Jerusalem guidelines [13], non-operative management with antibiotics is considered as a safe alternative to surgery in selected uncomplicated acute appendicitis patients without appendicolith (strength of recommendations: strong; 1A). The choice between antibiotic treatment and early appendectomy for uncomplicated appendicitis has been increasingly based on shared decision-making and patients’ choice on clinical practice [12]. However, a missed diagnosis of appendiceal perforation can lead to complications such as abscess formation and purulent peritonitis [14, 15]. The rupture rate of acute appendicitis is approximately 20–34% [16–19]. Patients who wish to avoid appendectomy must be aware of a recurrent risk of approximately 39% after 5 years. [9, 13] Each physician and surgeon should consider the advantages and disadvantages of each treatment option while managing acute appendicitis.

Many clinical scoring systems have been developed to evaluate acute appendicitis. Scoring systems widely used for clinically diagnosing acute appendicitis include the Alvarado score [20], Appendicitis Inflammatory Response score [21], Raja Isteri Pengiran Anak Saleha Appendicitis score [22], and adult appendicitis score (AAS) [23]. Moreover, compared with other imaging modalities, computed tomography (CT) demonstrated a higher sensitivity (98%) and specificity (97%) in detecting acute appendicitis [24–26]. Patients with an Alvarado score of 4–6 are recommended to undergo CT [27–29]. A recent large-scale international study [30, 31] has conducted the comparison of scoring systems that are used for the diagnosis of acute appendicitis, and it reveals AAS is the best performing score [30]. While an AAS of > 8 for women or an AAS of > 6 for men has higher probability of acute appendicitis [30], an AAS score of < 11 has very low risk of complicated appendicitis [23]. However, approximately 94% of patients with an AAS score of < 11 are in fact not the cases of acute appendicitis in this study [23]; naturally, these patients cannot develop complicated appendicitis. A more reliable model is required to identify patients with a higher risk of perforated appendicitis when they decide to receive treatment without surgery.

To the best of our knowledge, a total of eight models for predicting the risk of perforated appendicitis have been developed by Bröker et al. [32], Imaoka et al. [28], Khan et al. [16], Kim et al. [33], Kang et al. [34], Atema et al. [35], Avanesov et al. [36], and Kim et al. [37]. However, the number and types of factors included for predicting complicated appendicitis differ considerably among these eight models. For example, in the simplest model, which was developed by Bröker et al. [32], the following two factors were proposed for predicting cases of complicated acute appendicitis: an increased C-reactive protein (CRP) level and the abdominal pain duration. Furthermore, in the most complex model, which was developed by Atema et al. [35], both clinical factors and imaging features were used for predicting complicated acute appendicitis; they assigned a score of 0–22 and 0–19 points for CT and ultrasound findings, respectively. Thus, the application of this complex model would be time-consuming in clinical practice.

In this study, we compared and externally validated all currently available scoring systems used for identifying complicated acute appendicitis. A meta-analysis indicated that periappendiceal fat stranding (FS) had higher sensitivity (94%) than other CT features in predicting complicated acute appendicitis [1]. A study reported that the observation of FS on CT was associated with a tenfold higher likelihood of complicated appendicitis [37]. Therefore, we also developed an easy-to-use scoring model incorporating the three grades of FS observed on CT.

## Methods

### Study design and patients

This retrospective observational study, based on a prospective registry, was performed in the emergency department, Taipei Medical University Hospital, a tertiary referral and academic hospital with 750 beds in Taipei City, Taiwan. The purpose of building the registry was to develop a tool for tracking the clinical care and outcomes for patients presenting with acute abdomen in our emergency department. This registry system included information regarding patients’ demographic characteristics, physical examination findings, laboratory testing data, and reports on CT on arrival to emergency department; past history of medical comorbidities; operation note findings; and pathology reports. In this registry, data curation and verification were conducted by 3 physicians (HA Lin, HW Tsai, and CC Chao), and the protocol was developed by 2 physician (HA Lin, and SF Lin) in Taipei Medical University Hospital.

From our prospective registry, we retrieved the following data of consecutive patients who underwent treatment at the Department of Emergency Medicine, Taipei Medical University Hospital, Taipei, Taiwan, between January 1, 2015, and December 31, 2019: age, sex, body mass index (BMI), medical history, physical examination results, laboratory test results, and radiographic findings. In this study, patients with a confirmed diagnosis of acute appendicitis who (1) received CT in the emergency department, (2) underwent appendectomy, and (3) had pathology findings compatible with the clinical diagnosis of acute appendicitis were included. On the basis of operation and pathology findings, we categorized patients into uncomplicated and complicated acute appendicitis groups. Patients with (1) perforated appendicitis, and/ or (2) gangrenous appendicitis, and/ or (3) complications such as diffuse peritonitis and abscess formation were considered to have complicated acute appendicitis. We excluded patients who (1) were aged < 20 years, (2) were discharged against medical advice, (3) were treated conservatively without operation, (4) had final diagnosis rather than acute appendicitis, (5) had surgical and/or pathological findings were not compatible with acute appendicitis, (6) were pregnant and therefore did not receive CT scan, and (7) had missing data in our registry. This study was approved by the Joint Institutional Review Board (IRB) of Taipei Medical University (reference number: N201905057). The requirement of informed consent was waived by the IRB because the data used were anonymous and deidentified.

### CT Imaging and interpretation

**Fig. 1:**
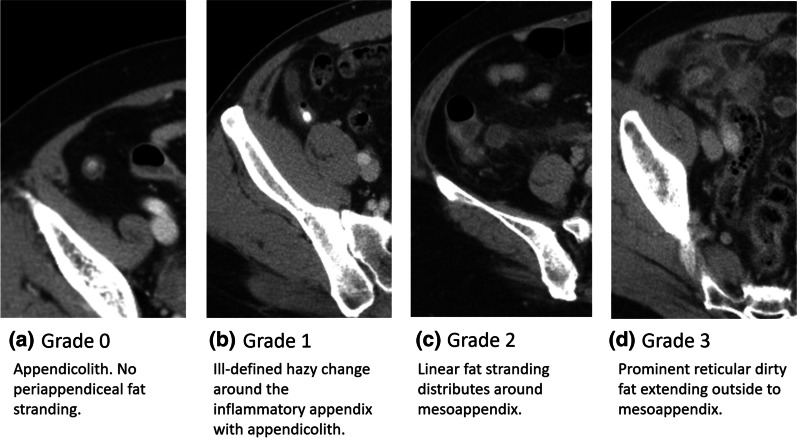
Grading of periappendiceal fat stranding

CT was performed in patients with acute appendicitis by using the 128-slice Somatom Perspective Scanner (Siemens, Germany). Scanning was performed from the top of the liver to the symphysis pubis with a 0.625-mm-thick spiral section. Patients were administered 95 mL of Optiray 350 contrast medium intravenously. CT scans were independently evaluated by observers who were blinded to the medical history of the patients. To verify the correctness of imaging data, one of the authors (HA Lin) repeatedly reviewed all original CT images. In accordance with Kim’s study [38], we used a 4-point scale for examining periappendiceal FS on CT (Fig. 1), wherein grades 0, 1, 2, and 3 indicated “definitely no sign of FS,” “mild FS of the adjacent fat (thickness < 2 mm),” “moderate FS of the adjacent fat confined to the mesoappendix,” and “severe FS extending outside the mesoappendix that is disproportionately greater than the degree of wall thickening,” respectively.

### Risk scoring models of complicated acute appendicitis

Table @@@3 summarizes the eight risk scoring models used for differentiating uncomplicated acute appendicitis from complicated acute appendicitis. Of the eight models, the original model developed by Bröker et al. [32] included the following continuous variables: the CRP level and abdominal pain duration. In addition, Bröker et al. [32] proposed cutoff values of ≥ 1 mg/dL for the CRP level and ≥ 2 days for the abdominal pain duration. We revised the cutoff of the CRP level to ≥ 3 mg/dL because a CRP level of 0–3 mg/dL is used as the reference limit in our and most other hospital laboratories. Furthermore, Khan et al. [16] used two continuous variables, namely age and abdominal pain duration, and one discrete variable, namely appendicolith (present or absent), in their model. We used the cutoff values of 40 and 60 years for age and ≥ 2 days for the abdominal pain duration. For the remaining six models, their original cutoff values for continuous variables were considered as their default values.

As a separate study, we performed an additional analysis to examine whether AAS could be used to predict complicated acute appendicitis. The original and modified versions of AAS systems were used (Additional file 1: Table S3). The only difference between the original and modified versions of AAS systems was the point assigned for a higher level of CRP.

### Statistical analysis

The general characteristics and clinical factors between the uncomplicated and complicated acute appendicitis groups were compared using Student’s *t* test for continuous variables and Pearson’s Chi-square or Fisher’s exact test for discrete variables. Simple and multivariate logistic regression models were employed to obtain the odds ratios (ORs), the area under the curve or *c* statistics of the receiver operating characteristic (ROC) curves, and their 95% confidence intervals (CIs). In the logistic regression model, the diagnosis of complicated or uncomplicated acute appendicitis was included as the dependent variable and any predictors or variables exhibiting significant differences between the uncomplicated and complicated appendicitis groups were included as independent variables. The optimal cutoff values for continuous variables that predicted complicated appendicitis were obtained using Youden’s *J* index (the maximal value of *J* = sensitivity + specificity − 1). To select appropriate variables in the multivariate logistic regression analysis for building our new models, we adopted two strategies: backward elimination (model 1) and stepwise selection (model 2). All statistical analyses were conducted using SAS, version 9.4. A two-tailed *P* value of < 0.05 indicated statistical significance.

### External validation

In the external validation study, we examined the performance of all the aforementioned eight models and our new developed models. The difference in the *c* statistics of the ROC curve between the model developed by Bröker et al. [32] (as a reference model) and the remaining models was determined by performing the integrated discrimination improvement (IDI) test. The goodness of fit of each model was examined by conducting the Hosmer–Lemeshow test (a model was considered to have good fitness when *P* > 0.05).

## Results

### Participants’ characteristics

We identified 583 consecutive patients suspected to have acute appendicitis in our prospective registry system for the period between January 1, 2015, and December 31, 2019.

Of the 181 patients who were excluded, 31 were aged < 20 years, 25 were discharged against medical advice, 20 were treated conservatively without operation, 54 had final diagnosis rather than acute appendicitis, 28 showed surgical and/or pathological findings which were not compatible with acute appendicitis, 2 received no CT scan due to pregnancy, and 21 had missing data in our registry. Finally, 402 patients with acute appendicitis were enrolled in this study (Table 1). Of them, 338 and 64 patients were categorized into the uncomplicated and complicated acute appendicitis groups, respectively (Additional file 2: Fig. S1). The mean ages of the uncomplicated and complicated appendicitis groups were 42.5 ± 16.5 and 49.0 ± 20.0 years, respectively (*P* = 0.0170). Compared with the uncomplicated appendicitis group, the complicated appendicitis group had a longer right lower quadrant (RLQ) pain duration (1.6 ± 1.0 vs. 2.5 ± 1.6 days, *P* < 0.0001), higher body temperature (36.8 ± 0.6 vs. 37.2 ± 0.8 °C, *P* = 0.0008), higher neutrophil to lymphocyte ratio (NLR; 8.9 ± 7.7 vs. 13.8 ± 0.7, *P* = 0.0008), and higher CRP level (2.9 ± 4.2 vs 12.8 ± 11.3, *P* < 0.0001). On CT imaging, a higher proportion of patients in the complicated acute appendicitis group exhibited appendicolith (27.9% vs. 48.4%, *P* = 0.0011), presence of ascites (13.6% vs. 29.7%, *P* = 0.0014), periappendiceal fluid (11.2% vs. 35.9%, *P* < 0.0001), intraluminal air (12.7% vs. 23.4%, *P* = 0.0253), extraluminal air (0.3% vs. 6.3%, *P* = 0.0026), and higher FS grades (grade scale of 1.0 ± 1.0 vs. 2.2 ± 0.9, *P* < 0.0001). In addition, pathology findings indicated that compared with the uncomplicated appendicitis group, the complicated acute appendicitis group demonstrated increased appendiceal width, gangrenous changes, and perforation as well as a longer hospital stay (2.5 ± 1.3 vs. 5.4 ± 3.8 days, *P* < 0.0001). However, no significant differences in sex, BMI, prior abdominal surgery, and blood pressure were noted between the two groups.

**Table 1:** Characteristics of patients with acute appendicitis (*N* = 402)

	Uncomplicated appendicitis (*N* = 338)	Complicated appendicitis (*N* = 64)	*P* value
Demographic factors			
Age (years)	42.5 ± 16.5	49.0 ± 20.0	0.0170*
Age groups (years)			0.0109*
20–25	53 (15.7%)	8 (12.5%)	
26–35	90 (26.6%)	13 (20.3%)	
36–45	69 (20.4%)	14 (21.9%)	
46–55	47 (13.9%)	3 (4.7%)	
56–65	39 (11.5%)	8 (12.5%)	
≥ 66	40 (11.8%)	18 (28.1%)	
Female (*n*/total *n*, %)	166 (49.1%)	35 (54.7%)	0.4134
BMI	23.5 ± 3.9	24.3 ± 4.1	0.1814
Clinical findings in ED			
Duration of RLQ pain (days)	1.6 ± 1.0	2.5 ± 1.6	< 0.0001*
Pain score (VAS)	5.4 ± 1.08	5.4 ± 1.9	0.9953
Previous abdominal surgery	53 (15.7%)	5 (78.8%)	0.1005
Duration of stay in ED (hour)	22.2 ± 11.1	21.4 ± 9.8	0.5706
Body temperature (°C)	36.8 ± 0.6	37.2 ± 0.8	0.0008*
Blood pressure			
Systolic	128.4 ± 19.1	129.7 ± 18.9	0.6217
Diastolic	77.1 ± 14.5	77.8 ± 12.5	0.7467
Mean arterial	94.2 ± 14.6	95.1 ± 13.6	0.6684
Laboratory factors in ED			
WBC count (10^3^ cells/μL)	13.7 ± 4.1	14.6 ± 4.4	0.1420
Neutrophil count (10^3^ cells/μL)	11.1 ± 4.0	12.2 ± 4.1	0.0593
Lymphocyte count (10^3^ cells/μL)	1.6 ± 0.7	1.3 ± 0.7	0.0025
NLR	8.9 ± 7.7	13.8 ± 0.7	< 0.0001*
Platelet (10^3^ cells/μL)	233.0 ± 54.4	240.5 ± 89.9	0.5181
CRP level (mg/dL)	2.9 ± 4.2	12.8 ± 11.3	< 0.0001*
Radiological findings of CT in ED			
Appendicolith (*N*)	94 (27.9%)	31 (48.4%)	0.0011*
Appendiceal diameter (cm)	10.96 ± 3.34	11.79 ± 4.24	0.1581
Cecum wall thickness (cm)	0.04 ± 0.19	0.10 ± 0.31	0.0865
Ascites (*N*)	46 (13.6%)	19 (29.7%)	0.0014*
Appendiceal hyperemia (*N*)	213 (63.0%)	41 (64.1%)	0.8737
Periappendiceal fluid (*N*)	38 (11.2%)	23 (35.9%)	< 0.0001*
Intraluminal air (*N*)	43 (12.7%)	15 (23.4%)	0.0253*
Extraluminal air (*N*)	1 (0.3%)	4 (6.3%)	0.0026*
Fat stranding (*N*)	213(63.0%)	61 (95.3%)	< 0.0001*
Fat stranding grades	1.0 ± 1.0	2.2 ± 0.9	< 0.0001*
Fat stranding grades classification			< 0.0001*
Grade 0	121 (35.8%)	3 (4.7%)	
Grade 1	111 (32.8%)	11 (17.2%)	
Grade 2	80 (23.7%)	22 (34.4%)	
Grade 3	26 (7.7%)	28 (43.8%)	
Pathological findings			
Appendiceal length (cm)	5.45 ± 1.63	5.22 ± 1.49	0.3059
Appendiceal width (cm)	0.95 ± 0.40	1.37 ± 0.78	< 0.0001*
Infiltration (*N*)	83 (24.6%)	20 (31.3%)	0.2607
Gangrenous (*N*)	0 (0%)	11 (17.2%)	< 0.0001*
Perforatation (N)	0 (0%)	27 (42.2%)	< 0.0001*
Hospitalized days (day)	2.5 ± 1.3	5.4 ± 3.8	< 0.0001*

*BMI* body mass index, *cm* centimeter, *CRP* C-reactive protein, *CT* computed tomography, *ED* emergency department, *NLR* neutrophil to lymphocyte ratio, *RLQ* right lower quadrant, *VAS* visual analog scale, *WBC* white blood cell

^*^Statistical significance (*P* < 0.05)

### Factors associated with complicated acute appendicitis (univariate analysis)

Clinical factors and radiographic features associated with complicated acute appendicitis are listed in Additional file 1: Table S1. The results of univariate analysis demonstrated that age, body temperature, RLQ pain duration, NLR, and CRP were significantly associated with complicated appendicitis. The cutoff values for these continuous variables were set according to Youden’s *J* index: age > 60 years (OR: 2.71, 95% CI 1.51–4.86, *P* = 0.0008), body temperature > 37.4 °C (OR: 2.18, 95% CI 1.49–3.20, *P* < 0.0001), NLR > 10 (OR: 2.78, 95% CI 1.61–4.78, *P* = 0.0004), RLQ pain ≥ 2 days (OR: 3.94, 95% CI 2.20–7.03, *P* < 0.0001), and CRP = 3.0–5.9 mg/dL (OR: 4.06, 95% CI 1.67–9.89, *P* < 0.0001) and ≥ 6.0 mg/dL (OR: 4.06, 95% CI 1.67–9.89, *P* < 0.0001). Furthermore, the CT findings of FS (OR: 11.93, 95% CI 3.67–38.83, *P* < 0.0001), ascites (OR: 2.68., 95% CI 1.44–4.20, *P* = 0.0018), appendicolith (OR: 2.43, 95% CI 1.41–4.20, *P* = 0.0014), intraluminal air (OR: 2.09, 95% CI 1.06–3.99, *P* = 0.0258), extraluminal air (OR: 22.40, 95% CI 3.25–441.96, *P* = 0.0058), and periappendiceal fluid (OR: 4.41, 95% CI 2.39–8.14, *P* < 0.0001) were strongly associated with complicated acute appendicitis.

### Developing scoring system models (multivariate analysis)

In the exploratory model, the variables that exhibited significance in the univariate analysis were used for multivariate logistic regression. Among these variables, several clinical characteristics—age, body temperature, and RLQ pain duration—and CT features—periappendiceal fluid, intraluminal air, and extraluminal air—were found to be significantly associated with complicated acute appendicitis (Additional file 1: Table S2).

**Fig. 2:**
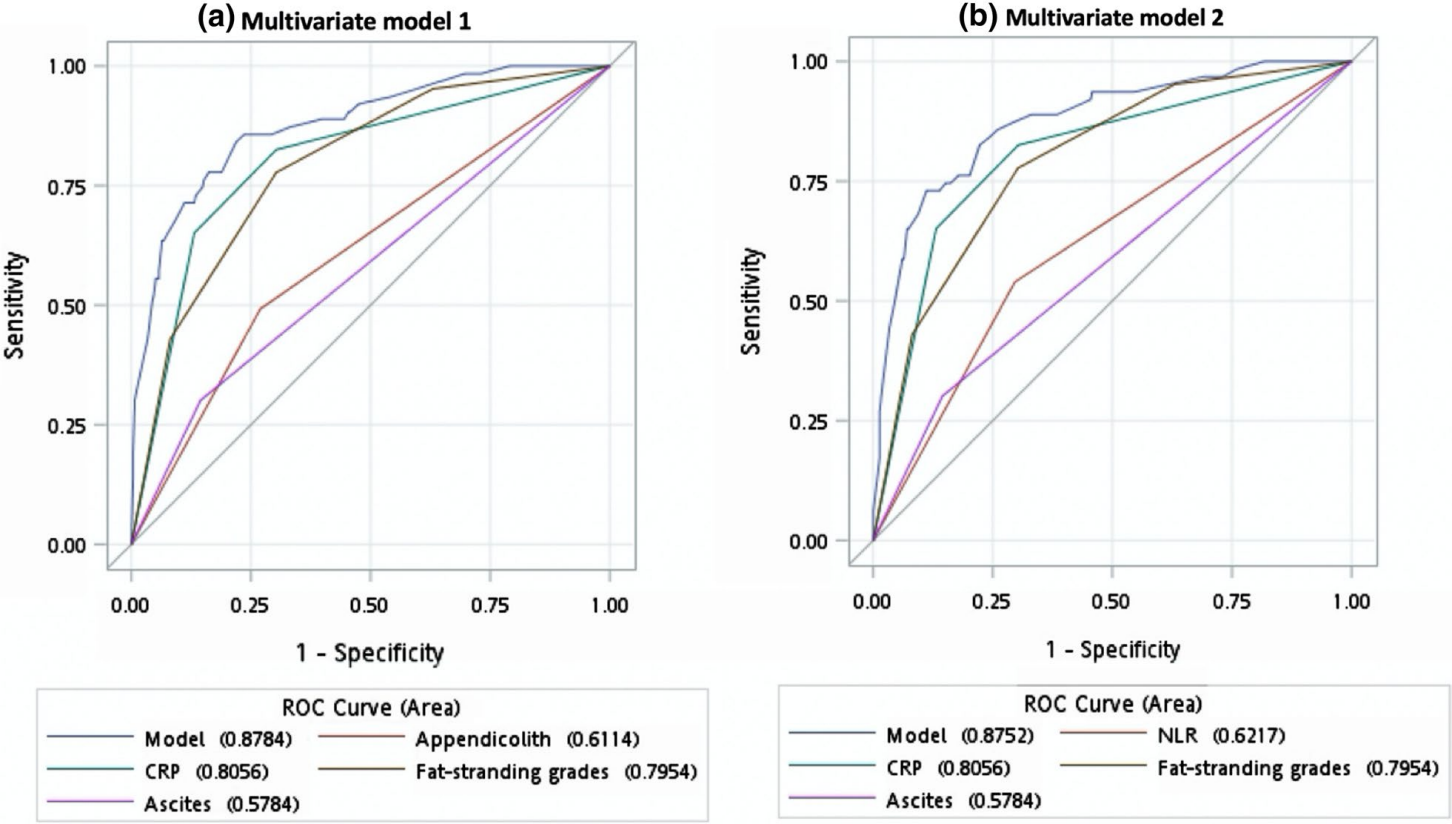
Receiver operating characteristic (ROC) curves for the multivariate logistic regression of **a** our model 1 and **b** our model 2

We developed a scoring system model by incorporating the three grades of FS (Table 2). In model 1 (variables selected through backward elimination), a CRP level of 3.0–5.9 mg/dL (OR: 3.58, 95% CI 1.33–9.59, *P* = 0.0114) and ≥ 6.0 mg/dL (OR: 11.61, 95% CI 4.95–27.21, *P* < 0.0001), grade 1 FS (OR: 4.26, 95% CI 1.08–16.74, *P* = 0.0381), grade 2 FS (OR: 6.02, 95% CI 1.56–22.78, *P* = 0.0083), grade 3 FS (OR: 18.44, 95% CI 4.70–72.36, *P* < 0.0001), appendicolith (OR: 2.94, 95% CI 1.43–6.03, *P* = 0.0179), ascites (OR: 2.68, 95% CI 1.19–6.07, *P* = 0.0032) strongly predicted complicated acute appendicitis. In addition, model 2 (variables selected through stepwise selection) showed a similar magnitude of association for the CRP level, three FS grades, ascites, and NLR > 10 (OR: 2.11, 95% CI 1.05–4.23, *P* = 0.0362). The *c* statistics for our models 1 and 2 are displayed in Fig. 2.

**Table 2:** Developing a scoring system for predicting complicated perforated appendicitis

Variables	Multivariate (model 1)		Multivariate (model 2) *c* statistics = 0.8752		Score assigned (range of 0–10) *c* statistics = 0.8784	
	OR (95% CI)	*P* value	OR (95% CI)	*P* value	Model 1	Model 2
NLR > 10			2.11 (1.05–4.23)	0.0362*	–	1
CRP (per mg/dL)					(0–3)	(0–3)
3.0–5.9 mg/dL	3.58 (1.33–9.59)	0.0114	3.32 (1.27–8.72)	0.0148*	2	2
≥ 6.0 mg/dL	11.61 (4.95–27.21)	< 0.0001*	9.97 (4.30–23.08)	< 0.0001*	3	3
Fat stranding (per grade)					(0–5)	(0–5)
Grade 1	4.26 (1.08–16.74)	0.0381*	4.08 (1.40–16.04)	0.0439*	3	3
Grade 2	6.02 (1.56–22.78)	0.0083*	6.15 (1.66–22.82)	0.0066*	4	4
Grade 3	18.44 (4.70–72.36)	< 0.0001*	18.29 (4.76–70.25)	< 0.0001*	5	5
Appendicolith on CT	2.94 (1.43–6.03)	0.0179*			1	–
Ascites on CT	2.68 (1.19–6.07)	0.0032*	2.83 (1.25–6.39)	0.0124*	1	1

*BMI* body mass index, *CI* confidence interval, *CRP* C-reactive protein, *CT* computed tomography, *NLR* neutrophil to lymphocyte ratio, *Ref.* reference group, *Temp.* temperature, *OR* odds ratio, *RLQ* right lower quadrant, *WBC* white blood cell, *VAS* visual analog scale

^*^Statistical significance (*P* < 0.05)

**Fig. 3:**
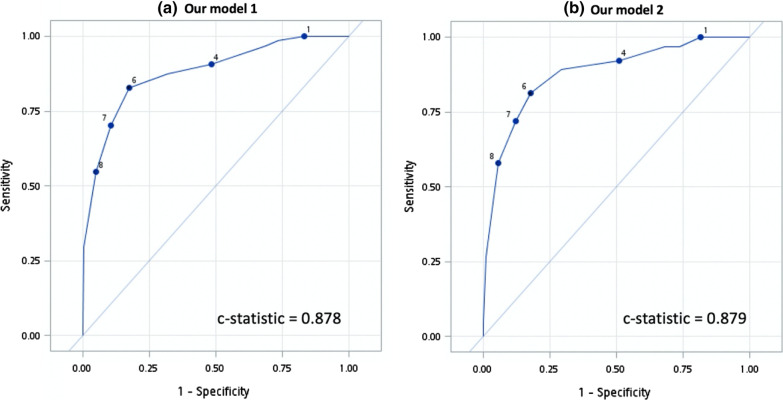
Receiver operating characteristic (ROC) curves indicated an optimal cutoff score of 6 for both our scoring systems: **a** model 1 and **b** model 2

We assigned scores for each variable according to the magnitude of the OR in predicting complicated acute appendicitis. In both models, CRP levels of 3.0–5.9 and ≥ 6.0 mg/dL were allocated a score of 2 and 3, respectively; grades 1, 2, and 3 FS were allocated scores of 3, 4, and 5, respectively; and ascites was assigned a score of 1. Furthermore, appendicolith and NLR were assigned a score of 1 in both model 1 and 2, respectively. Figure 3 reveals the ROC curves for our models 1 and 2. The optimal cutoff score of ≥ 6 (Table 3) exhibited a high sensitivity of 82.8% (95% CI 73.6%–92.1%) and a specificity of 82.8% (95% CI 78.5%–86.6%) in model 1 and 81.3% (95% CI 71.7%–90.8%) and 82.3% (95% CI 78.2%–86.3%) in model 2, respectively. The *c* statistics were 0.878 (95% CI 0.829–0.928) and 0.879 (95% CI 0.830–0.927) for models 1 and 2, respectively (Fig. 3).

**Table 3:** Scoring systems used for identifying complicated appendicitis

Models	Variables required	Points scored	Cutoff/total points	Sensitivity (95% CI)	Specificity (95% CI)	ROC *c* statistics (95% CI)
1. Bröker et al. [32]	CRP	≥ 3.0 (1)	2/2	64.1% (52.3–75.8%)	83.1% (79.1–87.1%)	0.778 (0.719–0.837)
	Duration of symptoms	≥ 2 days (1)				
2. Imaoka et al. [28]	Temp	≥ 37.4 °C (1)	1/3	84.9% (77.4–94.5%)	68.6% (63.7–73.6%)	0.800 (0.745–0.854)
	CRP	≥ 4.7 (1)				
	Periappendiceal fluid	Yes (1)				
3. Khan et al. [16]	Age	40–59 years (1)	2/4	73.4% (62.6–84.3%)	58.8% (53.6–61.1%)	0.694 (0.630–0.759)
		≥ 60 years (2)				
	Duration of symptoms	≥ 2 days (1)				
	Appendicolith	Yes (1)				
4. TH Kim et al. [33]	Appendiceal diameter	> 10 mm (1)	3/4	56.3% (44.1–68.4%)	86.1% (82.4–89.8%)	0.777 (0.718–0.835)
	Ascites	Yes (1)				
	Fat stranding	Yes (1)				
	CRP	> 5.0 mg/dL (1)				
5. Kang et al. [34]	Temp	≥ 37.9 °C (1)	4/8	60.9% (49.0–72.9%)	85.2% (81.4–89.0%)	0.772 (0.706–0.839)
	Abdominal pain score	4–6 (1), ≥ 7 (2)				
	WBC count	> 13,660/μL (1)				
	NLR	≥ 10.9 (1)				
	CRP	≥ 6.6 (3)				
6. Atema et al. [31]	Age	≥ 45 years (2)	7/22	76.6% (66.2–86.9%)	74.8% (70.2–79.5%)	0.826 (0.774–0.878)
	Temp	≤ 37.0 °C (0)				
		37.1–37.9 °C (2)				
		≥ 38.0 °C (4)				
	Duration of symptoms	≥ 48 h (2)				
	WBC count	> 13,000/μL (2)				
	CRP (mg/dL)	≤ 5.0 (0)				
		5.0–10.0 (1)				
		> 10.0 (2)				
	Extraluminal free air	Present (5)				
	Periappendiceal fluid	Present (2)				
	Appendicolith	Present (2)				
7. Avanesov et al. [36]	Age	≥ 52 years (1)	2/10	81.3% (71.7–90.8%)	69.2% (64.3–74.2%)	0.806 (0.749–0.862)
	Temp	≥ 37.5 °C (1)				
	Duration of symptoms	≥ 48 h (1)				
	Appendix diameter	≥ 14 mm (1)				
	Periappendiceal fluid	Present (2)				
	Extraluminal air present	Present (1)				
	Abscess	Present (3)				
8. HY Kim et al. [37]	Segmented neutrophils	≥ 81% (1)	3/6	64.1% (52.3–75.8%)	87.6% (84.1–91.0%)	0.838 (0.788–0.889)
	Contrast-enhancement of the appendiceal wall	Defect (1)				
	Abscess	Present (1)				
	Fat stranding	Moderate or severe (1)				
	Appendiceal diameter	≥ 10 mm (1)				
	Extraluminal air	Present (1)				
9. Model 1 (our model)	CRP (mg/dL)	3.0–5.9 (2)	6/10	82.8% (73.6–92.1%)	82.8% (78.5–86.6%)	0.878 (0.829–0.928)
		≥ 6.0 mg/dL (3)				
	Fat stranding	Grade 1 (3)				
		Grade 2 (4)				
		Grade 3 (5)				
	Appendicolith	Present (1)				
	Ascites	Present (1)				
10. Model 2 (our model)	CRP (mg/dL)	3.0–5.9 (2)	6/10	81.3% (71.7–90.8%)	82.3% (78.2–86.3%)	0.879 (0.830–0.927)
		≥ 6.0 mg/dL (3)				
	Fat stranding	Grade 1 (3)				
		Grade 2 (4)				
		Grade 3 (5)				
	NLR	> 10 (1)				
	Ascites	Present (1)				

*CRP* C-reactive protein, *CRP* C-reactive protein, *CT* computed tomography, *NLR* neutrophil to lymphocyte ratio, *WBC* white blood cell

### Validation of scoring systems for predicting complicated appendicitis

**Fig. 4:**
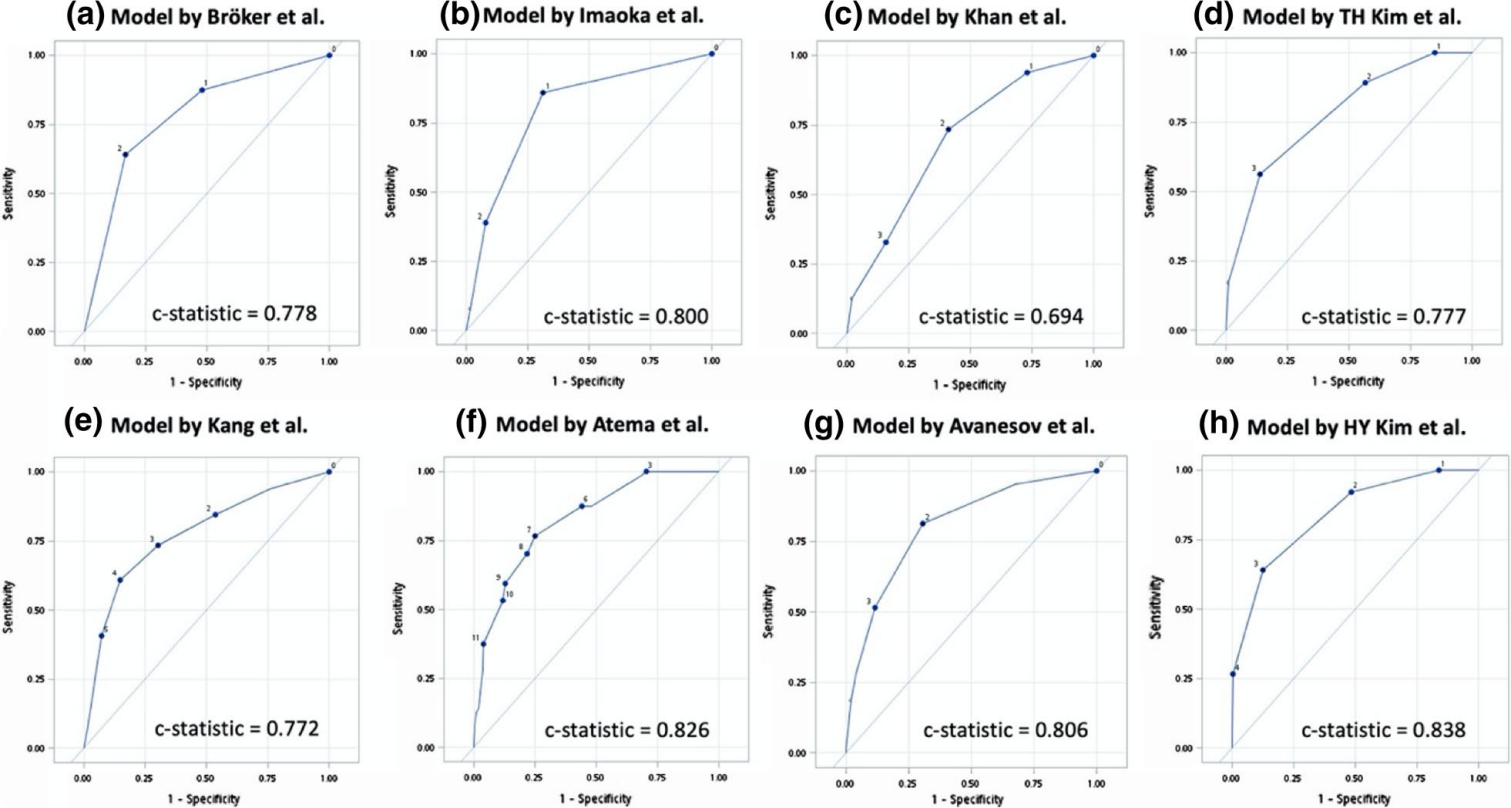
Receiver operating characteristic (ROC) curves of prior models predicting complicated acute appendicitis, including the models developed by **a** Bröker et al. **b** Imaoka et al. **c** Khan et al. **d** Kim et al. **e** Kang et al. **f** Atema et al. **g** Avanesove et al. and **h** Kim et al.

Table 3 lists the factors and imaging features used in each scoring system for predicting complicated appendicitis. In Table 3, models 1–8 are previously developed models, and models 9 and 10, respectively, correspond to model 1 and 2 that were developed in the current study. Among these scoring systems, model 1 developed by Bröker et al. was the simplest and included only two variables (CRP level and abdominal pain duration). This model with a total score of 2 exhibited a sensitivity of 64.1% (95% CI 52.3–75.8%) and a high specificity of 83.1% (95% CI 79.1–87.1%), with a *c* statistic of 0.778 (95% CI 0.719–0.837). Model 7 developed by Atema et al. [35] was the most complex. With an optimal cutoff of 22 points, this model showed a moderate sensitivity of 76.6% (95% CI 66.2–86.9%), a moderate specificity of 74.8% (95% CI 70.2–79.5%), and a *c* statistic of 0.826 (95% CI 0.774–0.878). Figure 2 presents the sensitivity and specificity determined by applying optimal cutoff values based on Youden’s *J* index for the remaining models (Fig. 2). The *c* statistic was 0.800 (95% CI 0.745–0.854) for model 2 developed by Imaoka et al. [28], 0.694 (95% CI 0.630–0.759) for model 3 developed by Khan et al. [16], 0.777 (95% CI 0.718–0.835) for model 4 developed by Kim et al. [33], 0.772 (95% CI 0.706–0.839) for model 5 developed by Kang et al. [34], 0.806 (95% CI 0.749–0.862) for model 7 developed by Avanesov et al. [36], and 0.838 (95% CI 0.788–0.889) for model 8 developed by Kim et al. [37]. The ROC curves of these models are shown in Fig. 4.

In a separate analysis of AAS, approximately > 85% of appendicitis patients in both groups of complicated and uncomplicated had high risk of appendicitis with AAS > 8 (Additional file 1: Table S4). In the original AAS, a *c* statistic for predicting complicated acute appendicitis was 0.512 (0.436–0.589), and the Youden’s index was at 13 with a poor sensitivity of 46.9% and a poor specificity of 54.4% (Additional file 1: Table S5). In the modified AAS, a *c* statistic was 0.625 (0.545–0.704), and the Youden’s index was at 15 with a poor sensitivity of 46.9% and a moderate specificity of 75.4%. The ROC curves for original and modified AAS models displayed poor performance in predicting complicated appendicitis (Additional file 2: Fig. S2).

### Comparison of scoring systems for predicting complicated appendicitis

As a separate validation study, we compared the performance of each scoring system in predicting complicated appendicitis (Table 4). Generally, all these models demonstrated an acceptable goodness of model fit except for the scoring system developed by Imaoka et al. [28]. All scoring systems exhibited significant ORs for predicting complicated acute appendicitis. By regarding model 1 developed by Bröker et al. [32] as the reference model, we observed significant differences among the *c* statistics of five scoring systems. Of these scoring systems, model 3 developed by Khan et al. [16] exhibited an inferior diagnostic accuracy (IDI: − 9.450%, *P* < 0.0001), whereas model 6 developed by Atema et al. [35] (IDI: 5.916%, *P* = 0.0248) and model 8 developed by Kim et al. [37] (IDI: 13.816%, *P* = 0.0006) demonstrated a superior diagnostic accuracy. In addition, our two models, model 9 (IDI: 18.292, *P* < 0.0001) and model 10 (IDI: 18.292, *P* < 0.0001), exhibited a considerably higher diagnostic accuracy for predicting complicated appendicitis, compared with model 1.

**Table 4:** Diagnostics for models used for discriminating complicated appendicitis

Models	Model odds ratio (per score increase)	*P* value	ROC *c* statistics	IDI (%)	*P* value	Hosmer–Lemeshow statistics (*χ*^2^)	*P* value
1. Bröker et al. [32]	4.18 (2.80–6.23)	< 0.0001*	0.778	Ref	Ref	0.467 (3 groups)	0.4942
2. Imaoka et al. [28]	3.65 (2.58–5.15)	< 0.0001*	0.800	0.919	0.7313	7.556 (3 groups)	0.0060*
3. Khan et al. [16]	1.92 (1.49–2.47)	< 0.0001*	0.694	− 9.450	< 0.0001*	3.200 (4 groups)	0.2019
4. TH Kim et al. [33]	3.61 (2.51–5.18)	< 0.0001*	0.777	1.811	0.5036	2.382 (4 groups)	0.3039
5. Kang et al. [34]	1.76 (1.51–2.09)	< 0.0001*	0.772	0.596	0.8310	2.767 (6 groups)	0.5975
6. Atema et al. [35]	1.44 (1.31–1.58)	< 0.0001*	0.826	5.916	0.0248*	7.289 (9 groups)	0.3995
7. Avanesov et al. [36]	2.29 (1.85–2.85)	< 0.0001*	0.806	3.511	0.2678	3.865 (5 groups)	0.2764
8. HY Kim et al. [37]	5.15 (3.44–7.71)	< 0.0001*	0.838	13.816	0.0006*	1.163 (4 groups)	0.5591
9. Lin (our model 1)	2.14 (1.79–2.56)	< 0.0001*	0.878	18.292	< 0.0001*	13.315 (10 groups)	0.1015
10. Lin (our model 2)	2.08 (1.75–2.46)	< 0.0001*	0.879	18.292	< 0.0001*	8.150 (8 groups)	0.2273

*IDI* integrated discriminatory improvement, *ROC* receiver of operating characteristics curve

## Discussion

In this study, we developed two scoring systems by including variables, namely the CRP level, CT features (three grades of FS and appendicolith), and ascites (model 1) or NLR > 10 (model 2), to distinguish between complicated and uncomplicated acute appendicitis. The scoring systems were based on biomarkers routinely collected in clinical practice. In our models, a score of 6 exhibited a high sensitivity and specificity (both > 80%) in predicting complicated appendicitis. Compared with prior models developed by Atema et al. [35] and Kim et al. [37] that exhibited *c* statistics of > 0.8, our scoring systems employed a lower number of variables and exhibited a higher diagnostic accuracy (*c* statistics = 0.878 and 0.879, respectively) with a stable goodness of fit.

In the meeting for the 2020 update of the WSES Jerusalem guidelines, there were debates [39] on the need of CT imaging for patients aged < 40 years having high probability of acute appendicitis according to the Alvarado score [20], Appendicitis Inflammatory Response score [21], and AAS [23] alone. In 2021, a large-scale study [40] reported the use of these scoring systems alone for selective CT should cause a great loss of accuracy (a loss of sensitivity to 49–81% and a loss of the specificity to 79–98%). When non-operative management with antibiotics for uncomplicated acute appendicitis has gradually become the standard management, CT imaging is considered a necessary tool to confirm the diagnosis before deciding to treat patients without surgery [39]. Moreover, a recent systematic review [39] has indicated that further research on evaluating which CT features help distinguish between uncomplicated and complicated acute appendicitis is warranted. Our study confirmed that the new developed models employing three grades of FS, in combination with biomarkers of CRP or NLR and CT features of appendicolith or ascites, were powerful to identify complicated acute appendicitis. On the other hand, models including no CT features, such as those developed by Bröker et al. [32] and Kang et al. [34], exhibited limited sensitivity.

Focal FS is generally an acceptable indicator for evaluating the severity of intraabdominal inflammation for surrounding organs [41, 42]. Back in 2003, a study [43] analyzing various CT features in acute appendicitis patients found that periappendiceal FS was one of the most distinguished features to discriminate appendicitis from alternative diagnoses with a reliable sensitivity of 87% and a specificity (74%). Because FS outweighs other radiographic features in differentiating between complicated and uncomplicated acute appendicitis [1, 42], we included FS in the new scoring system. Compared with prior models that also employed FS, our models were superior because they categorized FS into four grades (0–3). This grading system was also strongly supported by a recent study [44], which revealed attenuation of periappendiceal fat was significantly associated with the severity of appendicitis. Compared to patients with uncomplicated appendicitis, patients with complicated acute appendicitis exhibited higher CT number (or Hounsfield unit) of periappendiceal fat. [44] In our study, we assigned increasing scores in our new scoring system to different FS grades on the basis of their severity. Although model 4 developed by Kim et al. [33] included FS as a predictor, they did not categorize FS into different grades. Moreover, although model 8 developed by Kim et al. [37] also classified FS into different severity, in their final model, they included the moderate to severe grades of FS as a single predictor without assigning higher points for FS of greater severity. In addition to CT, an another recent study [45] has employed ultrasound to evaluate different grades of FS in appendicitis, and a higher grades of FS on ultrasound is found associated with higher risk of appendicitis. But further research on application of these grades in ultrasound examination is needed after considering its subjective nature.

CRP is a crucial laboratory test and the most widely used predictor for diagnosing complicated acute appendicitis [28, 32–35]. Prior models developed by Bröker et al. [32], Imaoka et al. [28], Kim et al. [33], Kang et al. [34], and Atema et al. [35] employed the CRP level as a marker. Our multivariate logistic regression performed using backward elimination and stepwise selection also retained the CRP marker. We speculate that the severity of inflammation, as demonstrated by FS on CT, plays a substantial role in determining the clinical course of acute appendicitis. In model 8 developed by Kim et al. [37], the substitution of CRP with a segmented neutrophil count of ≥ 81% also could effectively distinguish between complicated and uncomplicated acute appendicitis. In contrast to model 8 developed by Kim et al. [37], our new model 2 included the NLR rather than segmented neutrophils in the scoring system. Growing evidence has suggested that the NLR is not only a biomarker for inflammation but also a favorable indicator for the prognosis of cardiovascular disease [46], chronic kidney disease [47], malignancy [48], and even COVID-19 [49]. Therefore, we included NLR in our new model 2.

Our models were less susceptible to the confounding effect of the discrepancy in age. Although in model 8 developed by Kim et al. [37], patients were younger with a mean age of 15–44 years, in model 7 developed by Avanesov et al. [36], patients were older with a mean age of 56 years. Related studies [16, 35, 36] have used wider cutoff values for age, ranging from 40 to 60 years, for predicting complicated acute appendicitis. Although patients aged > 60 years are considered to have a higher risk of ruptured acute appendicitis, the effect of age was significantly attenuated in our multivariate analysis. Finally, we included various age groups in our models. The use of the CRP marker to reflect inflammation can be confounded in extremely young or old age groups because of the different visceral fat content in these groups [50–52]. Thus, models developed for distinguishing between complicated and uncomplicated acute appendicitis, such as the scoring systems developed by Atema et al. [35] and Kim et al. [37], should consider both laboratory and imaging features on CT.

Some factors could not effectively differentiate between complicated and uncomplicated acute appendicitis. No difference in the appendiceal rate was observed between male and female patients, and related studies have reported inconsistent results for sex. Some studies have indicated that a higher proportion of male patients developed appendiceal rupture [53, 54], whereas other studies have demonstrated that a higher proportion of female patients developed complicated appendicitis [55, 56]. Consistent with the findings of a previous study [57], BMI was not found to affect the risk of appendiceal rupture. Although fever is considered a hallmark of systemic inflammation, a considerably broad range of cutoff values for body temperature was used in prior scoring systems. For example, a cutoff value of ≥ 37.1 °C was used for body temperature in model 6 developed by Atema et al. [35], whereas a cutoff value of 37.9 °C was used in model 5 developed by Kang et al. [34]. We believe that body temperature as a marker can be affected by the environment, the use of antipyretics, and the availability of over-the-counter (OTC) antipyretics. Moreover, in Taiwan, OTC antipyretics, such as acetaminophen, are cheap and easily available to patients. A history of a longer abdominal pain duration was considered to be associated with appendiceal rupture [24]. This finding is based on the presumption that uncomplicated acute appendicitis will progress to rupture eventually. However, this presumption may not be completely correct because recent randomized controlled trials [6–9] and meta-analyses [10–12] have reported promising results for selected cases. This factor was also found to be attenuated in our multivariate analysis.

The findings of our validation study are consistent with those of the original analysis. For example, the *c* statistics reported by Kim et al. [37] were 0.80 (95% CI 0.77–0.83) and 0.81 (95% CI 0.77–0.85, based on their validation data set); these values are similar to the *c* statistic of 0.838 (0.788–0.889) determined for model 8 developed by Kim et al. [37] in the present study. Moreover, in accordance with the *c* statistic of 0.88 (95% CI 0.85–0.92) reported by Atema et al. [35] for their model that included clinical and CT features, we determined a *c* statistic of 0.826 (95% CI 0.774–0.878) for model 6 developed by Atema et al. [35]. These findings suggest the applicability of our models to other populations.

In a separate analysis, AAS showed poor predictability for complicated appendicitis. We considered AAS, like the Alvarado score and Appendicitis Inflammatory Response score, was developed for assisting the diagnosis of acute appendicitis rather than for discriminating between complicated and uncomplicated appendicitis. These scores focused on the findings of physical examination and serum biomarkers, and did not assess the CT features. Compared to other models with CT features, AAS showed poor performance to discriminate between complicated and uncomplicated appendicitis. Moreover, we have tried to modify the AAS by assigning higher score for a higher CRP level since the original AAS assigning a lower score for CRP. Although this modification had increased the specificity from 54.4 to 75.4%, the modified AAS was still not practical to identify complicated appendicitis (Additional file 1: Table S5). This analysis supported the importance of the use of CT features to discriminate between complicated and uncomplicated acute appendicitis.

Although we comprehensively investigated demographic factors, symptoms and signs, laboratory test results, and CT features, this study has some limitations. First, this was a retrospective observational study; thus, some residual factors such as the medication history and medical comorbidities were not fully considered. Second, in prior studies, the definition of complicated acute appendicitis was not universally consistent. Some studies have defined complicated appendicitis on the basis of surgical reports, whereas others have employed pathohistological results. Similar to our analysis, some studies have also defined a case considering both surgical and pathological results. Third, we did not adopt low-dose protocols for the CT scan. A randomized controlled trial showed that low-dose CT was noninferior to standard-dose CT [58] with respect to negative appendectomy rates. But this low-dose CT protocol has caused a greater noise in imaging. Although recently studies [59, 60] have proposed the new technique adaptive statistical iterative reconstruction to reduce the imaging noise for low-dose CT, this technique was unavailable to us during the study period. We considered higher image quality was necessary for applying a 4-point scale to examine periappendiceal FS on CT. Fourth, patients aged < 20 years were excluded in our analysis. In Taiwan, the age of majority is set at 20 year old. Patients who aged < 20 years need the consent from their parent or legally appointed guardians to make a medical decision. While most pediatric patients who agreed to receive surgery were transferred to Taipei Municipal Wan-Fang Hospital, pediatric patients who received medical treatment without surgery still stayed in our hospital. The two hospitals are within a short distance of 6 km, belonging to the same medical system, and are both managed by Taipei Medical University. Since our registry had no records in Taipei Municipal Wan-Fang Hospital, we excluded patients aged < 20 years to avoid selection bias. Lastly, all models could not completely guarantee the clinical course for patients. Nevertheless, our analysis and scoring systems can assist physicians and surgeons in effectively distinguishing between uncomplicated and complicated acute appendicitis preoperatively, thus helping them make a more precise decision regarding the timing of appendectomy.

## Conclusions

In conclusion, our developed models and prior scoring systems developed by Atema et al. [35] and Kim et al. [37] were validated to have a high diagnostic accuracy. However, our two models employ the lowest number of variables and can thus help rapidly distinguish between complicated and uncomplicated appendicitis in clinical practice. This differentiation can help patients with uncomplicated appendicitis avoid unnecessary surgery and subsequent complications.

## Supplementary Information

**Additional file 1**. Supplemental Tables.

**Additional file 2**. Supplemental Figures.

## Data Availability

Although an identified and anonymous data set was used, data cannot be shared publicly due to legal restrictions imposed by the government of Taiwan on the distribution of the “Personal Information Protection Act.” Data are only available from the formal proposal to Department of Emergency, Taipei Medical University Hospital, Taipei, Taiwan. The contact information was as follows: No. 252, Wuxing St, Xinyi District, Taipei City, 110, Taiwan (R.O.C.)
