# Supplementary material for: Periappendiceal fat-stranding models for discriminating between complicated and uncomplicated acute appendicitis: a diagnostic and validation study
Source: World J Emerg Surg. 2021 Oct 13;16:52. doi: 10.1186/s13017-021-00398-5 (PMC8511616; doi:10.1186/s13017-021-00398-5)
Supplement: Supplementary file 1 — Additional file 1. Supplemental Tables. [file 13017_2021_398_MOESM1_ESM.pdf]

Supplemental Table S1. Univariate analysis for predicting complicated perforated appendicitis

Variables	OR (95% CI)	*P value*
Age (per 10 years)	1.23 (1.06–1.43)	0.0063*
Age > 60 years	2.71 (1.51–4.86)	0.0008*
Sex (female vs. male)	1.25 (0.73–2.15)	0.4140
BMI (per kg/m^2 increase)	1.05 (0.98–1.12)	0.1818
Body Temp. (per °C)	2.18 (1.49–3.20)	<0.0001*
Body Temp. > 37.4 °C	2.29 (1.17–4.32)	0.0126*
WBC count (10^3 cells/μL)	1.05 (0.98–1.12)	0.1427
Neutrophil (10^3 cells/μL)	1.00 (1.00–1.00)	0.0607
NLR	1.05 (1.02–1.08)	0.0014*
NLR > 10	2.78 (1.61–4.78)	0.0002*
Platelet (10^3 cells/μL)	1.00 (1.00–1.01)	0.3687
RLQ pain days (per day)	1.63 (1.35–2.00)	<0.0001*
RLQ pain ≥ 2 days	3.94 (2.20–7.03)	<0.0001*
CRP (per mg/dL)	1.20 (1.14–1.26)	<0.0001*
3.0–5.9 mg/dL	4.06 (1.67–9.89)	0.0020*
≥ 6.0 mg/dL	19.78 (9.36–41.81)	<0.0001*
Fat stranding on CT	11.93 (3.67–38.83)	<0.0001*
Fat stranding (per grade)	3.39 (2.43–4.73)	<0.0001*
Grade 1	4.00 (1.09–14.70)	<0.0001*
Grade 2	11.09 (3.21–38.29)	<0.0001*
Grade 3	43.44 (12.27–153.71)	<0.0001*
Appendicolith on CT	2.43 (1.41–4.20)	0.0014*
Ascites on CT	2.68 (1.44–4.98)	0.0018*
Periappendiceal fluid	4.41 (2.39–8.14)	<0.0001*
Intraluminal air on CT	2.09 (1.06–3.99)	0.0285*
Extraluminal air on CT	22.4 (3.25–441.96)	0.0058*
Pain score (per VAS)	1.00 (0.86–1.16)	0.9953

Abbreviations: BMI, body mass index; CI, confidence interval; CRP, C-reactive protein; CT, computed tomography; NLR, neutrophil to lymphocyte ratio; Ref. reference group; Temp., temperature; OR, odds ratio; RLQ, right lower quadrant; WBC, white blood cell; VAS, visual analog scale

*Statistical significance ($P < 0.05$).

Supplemental Table S2. Initial multivariate logistic regression model

	Multivariate<br>*c* statistics = 0.8858	
Variables	OR (95% CI)	*P value*
Age > 60 years	1.86 (0.80–4.33)	0.1511
Body Temp. > 37.4 °C	0.58 (0.24–1.41)	0.2327
NLR > 10	2.01 (0.96–4.21)	0.0661
RLQ pain ≥ 2 days	1.21 (0.54–2.70)	0.6496
CRP (per mg/dL)		
3.0–5.9	3.66 (1.33–10.05)	0.0120*
≥ 6.0	9.18 (3.49–24.15)	<0.0001*
Fat stranding (per grade)		
Grade 1	3.31 (0.83–13.18)	0.0899
Grade 2	4.57 (1.18–17.65)	0.0277*
Grade 3	13.47 (3.24–55.97)	0.0003*
Appendicolith (yes vs. no)	2.72 (1.26–5.86)	0.0105*
Ascites (yes vs. no)	2.58 (1.11–6.02)	0.0298*
Periappendiceal fluid (yes vs no)	1.67 (0.72–3.87)	0.2170
Intraluminal air (yes vs. no)	1.15 (0.43–3.07)	0.7896
Extraluminal air (yes vs. no)	2.49 (0.24–25.70)	0.4023

CI, confidence interval; NLR, neutrophil-lymphocyte ratio; RLQ, right lower quadrant; CRP, C-reactive protein

Supplemental Table S3. Original and modified points in Adult Appendicitis Score.

Variables required	Condition	Original score	Modified score
Symptoms and findings			
Pain in RLQ	Present	2	2
Pain relocation	Present	2	2
RLQ tenderness	Women, aged 16–49 years	1	1
	All other patients	3	3
Guarding	Mild	2	2
	Moderate	4	4
Laboratory tests			
Blood leukocyte count	≥ 7.2 and <10.9 (×10^9)	1	1
	≥ 10.9 and <14.0 (×10^9)	2	2
	≥ 14.0 (×10^9)	3	3
Proportion of neutrophil	≥ 62 and < 75 (%)	2	2
	≥ 75 and < 83 (%)	3	3
	≥ 83 (%)	4	4
CRP, symptoms < 24 h	≥ 0.4 and < 1.1 (mg/dl)	2	2
	≥ 1.1 and < 2.5 (mg/dl)	3	3
	≥ 2.5 and < 8.3 (mg/dl)	5	5
	≥ 8.3 (mg/dl)	1	6*
CRP, symptoms < 24 h	≥ 1.2 and < 5.3 (mg/dl)	2	2
	≥ 5.3 and < 15.2 (mg/dl)	2	4*
	≥ 15.2 (mg/dl)	1	5*

*The different points are assigned between original and modified scoring systems.

CRP, C-reactive protein; RLQ, right lower quadrant;

Supplemental Table S4. Adult Appendicitis Score in Enrolled Participants (*N* = 402)

	Uncomplicated appendicitis (*N* = 338)	Complicated appendicitis (*N* = 64)	*P* value
Original AAS	12.1 ± 3.4	12.0 ± 3.3	0.7736
†Original AAS > 8	286/338 (84.6%)	56/64 (87.5%)	0.5526
Modified AAS	12.5 ± 3.2	13.9 ± 3.4	0.0016*
†Modified AAS > 8	298/338 (84.1%)	60/64 (93.8%)	0.1895

AAS, adult appendicitis score.

*Statistical significance for $P < 0.05$.

†An AAS score of > 8 was considered with high probability of acute appendicitis.

Supplemental Table S5. Original and modified Adult Appendicitis Score used for identifying complicated appendicitis

Models	Cutoff/total points	Sensitivity (95% CI)	Specificity (95% CI)	ROC *c* statistics (95% CI)
Original AAS	13/23	46.9% (34.7–59.1%)	54.4% (49.1–59.8%)	0.512 (0.436–0.589)
Modified AAS	15/24	46.9% (34.7–59.4%)	75.4% (70.9–80.0%)	0.625 (0.545–0.704)

AAS, adult appendicitis score; CI, confidence interval; ROC, receiver of operating characteristics curve.
