# Supplementary material for: Periappendiceal fat-stranding models for discriminating between complicated and uncomplicated acute appendicitis: a diagnostic and validation study
Source: World J Emerg Surg. 2021 Oct 13;16:52. doi: 10.1186/s13017-021-00398-5 (PMC8511616; doi:10.1186/s13017-021-00398-5)
Supplement: Supplementary file 2 — Additional file 2. Supplemental Figures. [file 13017_2021_398_MOESM2_ESM.pdf]

Supplemental Figures

Number of Figures: 2

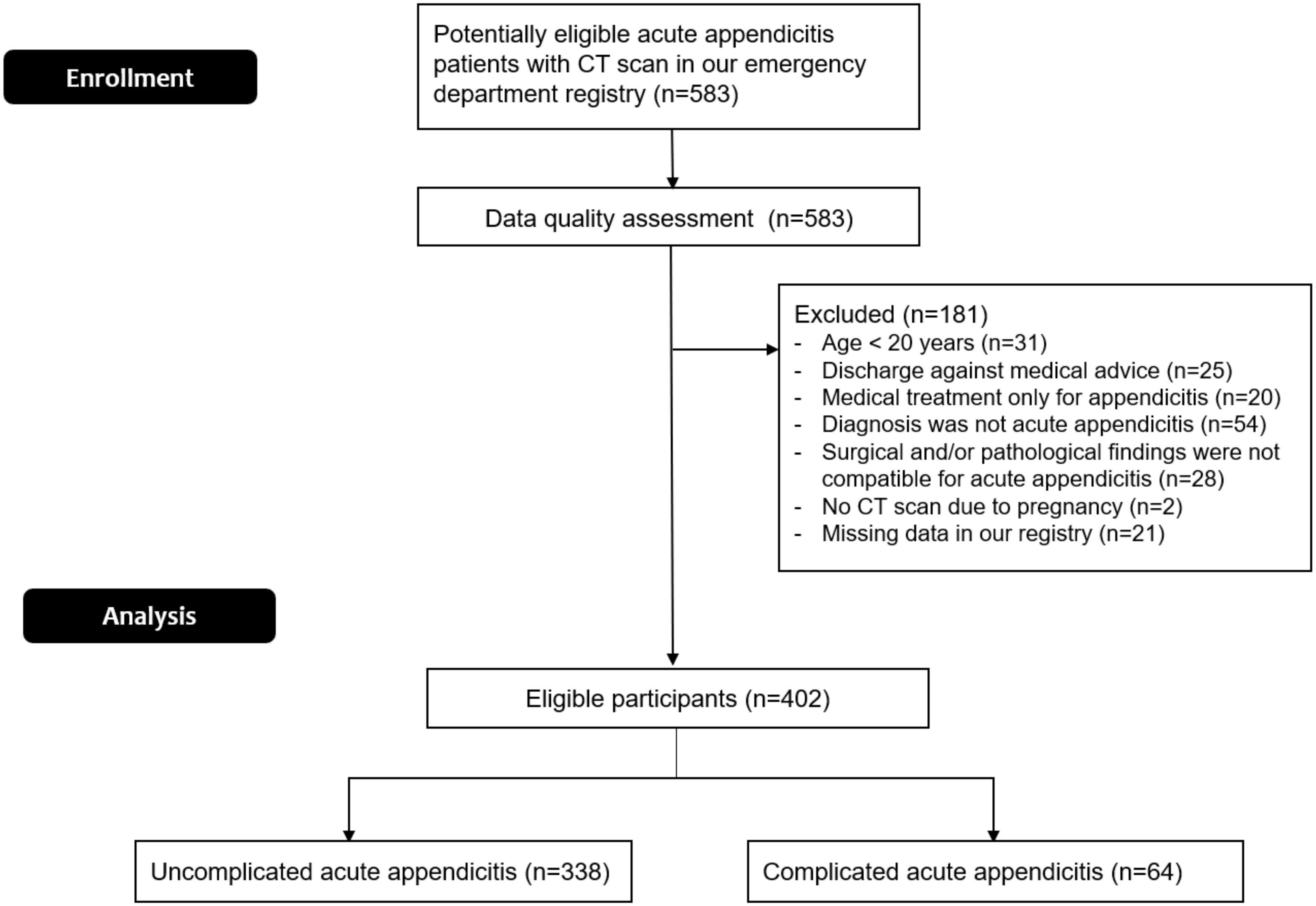

CT, computed tomography

Online Figure S1. Flow diagram of the study.

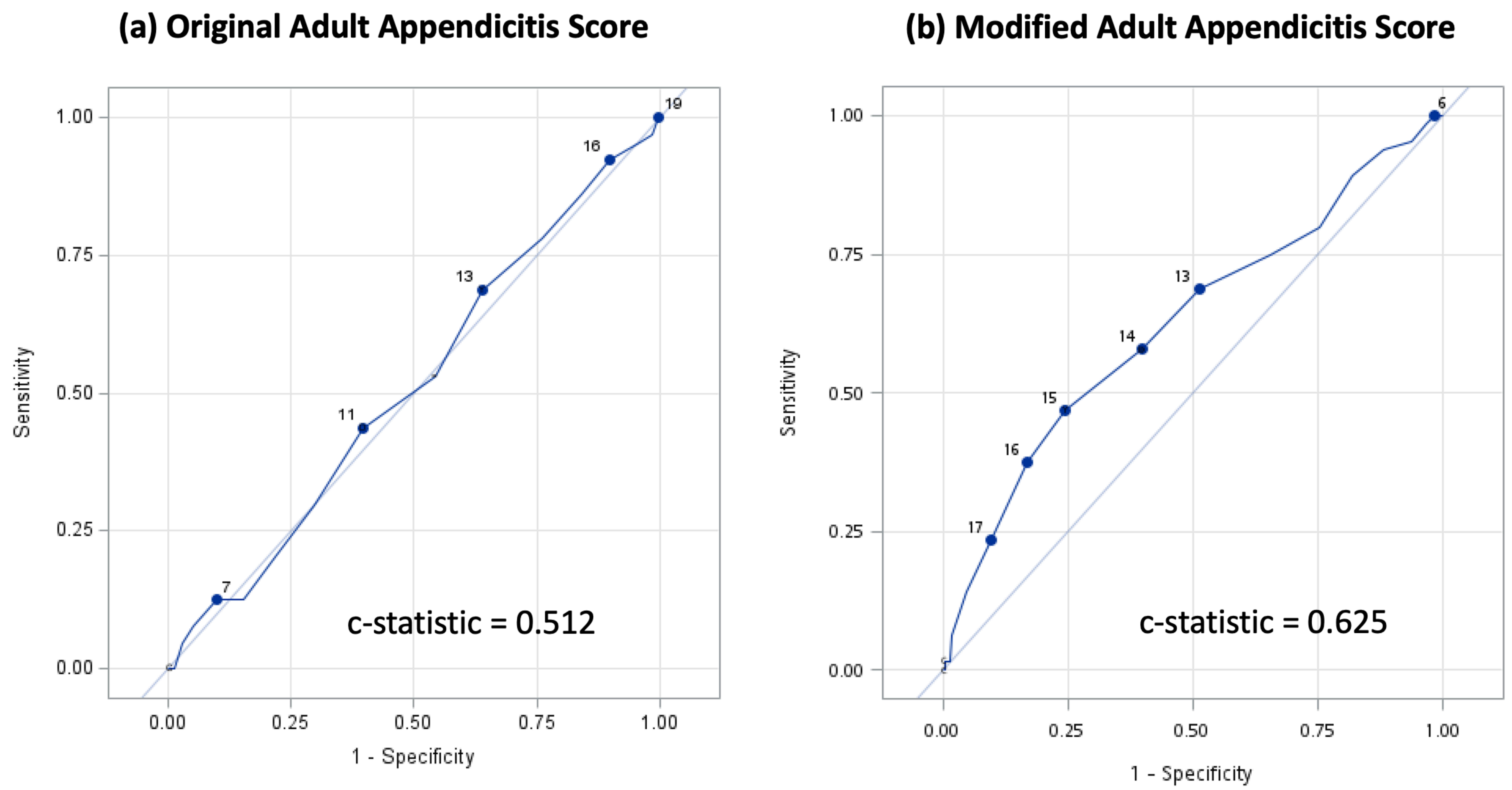

Online Figure S2. Receiver of operating characteristic curves of the Adult Appendicitis Score (AAS) for predicting complicated acute appendicitis.
